# Mindfulness-Based Stress Reduction Health Insurance Coverage: If, How, and When? An Integrated Knowledge Translation (iKT) Delphi Key Informant Analysis

**DOI:** 10.1007/s12671-024-02366-x

**Published:** 2024-05-17

**Authors:** Hannah E. Frank, Ariana Albanese, Shufang Sun, Frances Saadeh, Blair T. Johnson, A. Rani Elwy, Eric B. Loucks

**Affiliations:** 1 Department of Psychiatry and Human Behavior, The Warren Alpert Medical School, Brown University, Providence, RI USA; 2 Department of Behavioral and Social Sciences, School of Public Health, Brown University, Providence, RI USA; 3 Mindfulness Center, Brown University, Providence, RI USA; 4 School of Professional Studies, Brown University, Providence, RI USA; 5 Department of Psychological Sciences, University of Connecticut, Storrs, CT USA; 6 Center for Healthcare Organization and Implementation Research, VA Bedford Healthcare System, Bedford, MA USA; 7 Department of Epidemiology, School of Public Health, Brown University, Providence, RI 02910 USA

**Keywords:** Implementation science, Mindfulness-Based Stress Reduction, Delphi, Health insurance, Policy

## Abstract

**Objectives:**

Hundreds of trials have evaluated Mindfulness-Based Stress Reduction (MBSR), but in the United States, it is generally not covered by health insurance. Consequently, the aims were to identify the following: (1) key questions to make decisions about *if*, *how*, and *when* MBSR should be covered by health insurance; (2a) barriers and (2b) facilitators to understand and resolve for MBSR to be covered by health insurance; and (3) highest priority evidence needed to inform health insurance coverage decisions.

**Methods:**

Key informants (*n* = 26) included health insurers, healthcare administrators, policymakers, clinicians, MBSR instructors, and MBSR students. An initial pool of items related to the study aims was generated through qualitative interviews. Through the Delphi process, participants rated, discussed, and re-rated each item’s relevance. Items were required to reach a consensus of ≥ 80% agreement to be retained for final inclusion.

**Results:**

Of the original 149 items, 42 (28.2%) met the ≥ 80% agreement criterion and were retained for final inclusion. The most highly rated items informing whether MBSR should be covered by health insurance included research demonstrating that MBSR works and that it is not harmful. The most highly rated barriers to coverage were that MBSR is not a medical treatment and patient barriers to attendance. Highly rated facilitators included the potential of MBSR to address common mental health and psychosomatic problems. Finally, understanding what conditions are effectively treated with MBSR and the impact of MBSR on stress were rated as the highest priority evidence needed to inform health insurance coverage decisions.

**Conclusions:**

Findings highlight priorities for future research and policy efforts to advance health insurance coverage of MBSR in the United States.

**Supplementary Information:**

The online version contains supplementary material available at 10.1007/s12671-024-02366-x.

A major challenge in health research, and mindfulness research, is the “implementation cliff” (Weisz et al., 2013, p. 59). For example, intervention benefits typically drop when programs are scaled up (Onken et al., 2014). It takes an average of 17 years for 14% of research evidence to move from discovery to clinical implementation (Green et al., 2009). Furthermore, only one in five evidence-based interventions make it through to routine clinical practice (Kilbourne et al., 2020). Health insurance coverage of behavioral health interventions can be difficult to attain. Mindfulness-Based Stress Reduction (MBSR) is one behavioral health intervention that has witnessed over four decades of development since its inception in 1979 and subsequent scientific testing (Kabat-Zinn, 1982). Now, hundreds of randomized controlled trials have favorably evaluated MBSR, but it is largely not covered by health insurance in the United States (Goldberg et al., 2022; Goyal et al., 2014a).

MBSR is a standardized mindfulness-based program that was developed within a medical framework; it is a type of participatory medicine that takes place in group settings that challenges class members to discover and tap their personal resources for learning, healing, and transformation (Crane et al., 2017; Kabat-Zinn, 2013; Stahl & Goldstein, 2010). Standard MBSR is scheduled over eight weekly sessions lasting 2.5 hr, with a day-long retreat partway through the course, and about 45 min of guided mindfulness meditation home practice per day. MBSR is envisioned as being complementary and integrative with other evidence-based interventions (e.g., behavioral therapy, drugs), though few studies have directly evaluated its delivery in conjunction with other approaches (Lenze et al., 2022).

Because the number of people regularly meditating in the United States (US) recently doubled (Nahin et al., 2024) coupled with an evidence base favoring MBSR (de Vibe et al., 2017; Goldberg et al., 2022; Goyal et al., 2014a), there is increasing demand from health insurers, clinicians, and policymakers for health insurance coverage for MBSR as a standalone service. For example, leaders within the U.S. Veterans Health Administration asked the Department of Veterans Affairs (VA) Evidence Synthesis Program to undertake a systematic review examining the effects of MBSR on a range of health and mental health outcomes (Hempel et al., 2014). Similarly, the U.S. Agency for Healthcare Research and Quality (AHRQ) contracted a systematic review on meditation programs that included MBSR (Goyal et al., 2014a, 2014b). Most of the outcomes (e.g., depression, anxiety) in these two reviews were promising but inconclusive due to limited numbers of methodologically high-quality studies.

Evidence on the efficacy of MBSR has also been examined in meta-analyses. A Campbell systematic review with a meta-analysis of 101 MBSR trials (*N* = 8135) available through 2015 showed evidence in favor of impacts of MBSR on anxiety, depression, stress, quality of life, and physical functioning compared to inactive controls (de Vibe et al., 2017); the evidence was moderately strong based on the Grading of Recommendations Assessment, Development and Evaluation (GRADE) system. Another meta-analysis on MBSR for lower back pain found that MBSR reduced pain intensity within 8-week post-baseline compared to usual care, although longer-term effects were uncertain because only a few relevant studies were available (Anheyer et al., 2017). Effect sizes for MBSR versus control groups are typically in the medium effect size range when compared to inactive controls, and in the small effect size range when compared to active controls. These effect sizes are typical for effective behavioral medicine interventions and can have important clinical implications. Overall, the number of high-quality MBSR studies were limited in the Campbell review, which resulted in the “moderate” rather than “strong” level of evidence using the GRADE system (de Vibe et al., 2017).

Given that many of these reviews and meta-analyses are now dated and that high-quality MBSR clinical trials continue to be published, there is need for updated evidence syntheses. We use the term “evidence synthesis” to refer to the compilation of data from a variety of sources, including data from the research literature *and* input from key informants with relevant knowledge. It is rare for key informants to be involved in research projects from start to finish (Marquez et al., 2018; Redman et al., 2015; Soobiah et al., 2019; Tricco et al., 2018), yet the field of implementation science highlights the importance of doing so.

In the case of MBSR, key informants include policymakers, health insurers, healthcare administrations, clinicians, patients, MBSR students, and MBSR instructors. Input from these key informants ensures that investigators are pointed in a direction that will address the most important questions that inform MBSR health insurance coverage. Indeed, estimates show that 85% of investment in health and biomedical research is wasted every year due to redundancies, failures to address priorities based on key informants’ needs (particularly end-users), poor research methods, and incomplete reporting of study findings (Chalmers & Glasziou, 2009; Chalmers et al., 2014; Macleod et al., 2014; Tricco et al., 2018). Accordingly, scientists recommend integrated knowledge translation (iKT), where researchers and knowledge users (e.g., policymakers, health insurers, clinicians, patients) co-create knowledge, using tools such as the SPIRIT (Supporting Policy In health with Research: an Intervention Trial) Action Framework (Marquez et al., 2018; Redman et al., 2015; Soobiah et al., 2019; Tricco et al., 2018), described in more detail in the “Methods” section below. To our knowledge, no prior MBSR evidence synthesis has used an iKT approach. A goal of this project is to provide key informant recommendations on the next generation of clinical trials and evidence syntheses for MBSR. In addition, the current study considers the pragmatics of increasing access to MBSR via health insurance coverage.

What is needed for MBSR to be reimbursed by health insurance and to be billed as a standalone service (i.e., with its own billing code)? This question is unanswered in the scientific literature, particularly by key informants such as health insurers, policymakers, clinicians, patients, and MBSR teachers. Although MBSR is subsidized by health insurance in some countries (e.g., Singapore, Germany, Switzerland), it is typically not in the United States. One exception in the United States is through the Veterans Health Administration Directive 1137, which includes eight complementary and integrative health therapies, including mindfulness, in its standard medical benefits package for all Veterans receiving care in its national healthcare system (Kligler et al., 2022; United States Department of Veterans Affairs, 2022). The Veterans Health Administration is both a health system and a payer of services, providing care to over 9 million U.S. Veterans per year. Most other health insurers in the United States and in most other nations do not currently cover MBSR through health insurance.

The reasons for this lack of health insurance coverage are unclear but may be related to inadequate evidence syntheses on health effects of MBSR at a sufficient study quality level to drive policy change. Furthermore, there may be concern about participant burden. As noted above, MBSR involves 2.5-hr weekly classes once a week for 8 weeks plus an approximately 7.5-hr-long retreat; it may be more time than some patients are willing or able to spend taking the program. Some licensed clinicians bill for MBSR as “group therapy,” but this method of billing restricts compensated time to 90 min, meaning that the full MBSR content cannot be delivered within the allowable time. There may also be a lack of advocacy for health insurance coverage, as there are relatively few active advocates for it in the United States, and no entity seems likely to reap large economic benefits from health insurance coverage for MBSR. The lack of health insurance coverage for MBSR creates disparities in who may access and benefit from this program, hindering it from benefiting the greater public and particularly populations that may bear significant burdens of physical and mental health concerns (e.g., individuals with low incomes; people from racial/ethnic minoritized backgrounds; Cummings et al., 2017; Mongelli et al., 2020). It is important to assess the reasons for the lack of health insurance coverage for MBSR, and what would be needed for it to be provided.

The current study is the initial phase of a 5-year research project that aims to systematically examine the ways in which MBSR can become a covered practice by US health insurers. As such, the aims of this phase were to conduct formative qualitative interviews with health insurers, healthcare administrators, policymakers, clinicians, patients, and MBSR teachers to understand: (1) their questions about *whether*, *how*, and *when* MBSR should be covered by health insurance; (2a) perceived barriers and (2b) facilitators that are important to understand, resolve, and build upon in order for MBSR to be covered by health insurance; and (3) their beliefs about the highest priority evidence needed on MBSR’s effectiveness and cost-effectiveness to inform their decision-making. Answers to these questions can inform researchers who are identifying the most important hypotheses to test in MBSR clinical trials, systematic reviews, and meta-analyses that respond to societies’ greatest needs and opportunities. Findings from this study can also inform position statements from professional associations who inform policy and actions by decision makers on health insurance coverage of MBSR.

## Method

### Participants

Participants became members of an advisory board (*n* = 26) that was convened for the study. Members included MBSR experts (e.g., MBSR education leaders; clinicians and patients who have studied MBSR) and non-experts who had expertise in other key domains (e.g., health insurance and healthcare organization representatives, policymakers, and clinicians). Participants were recruited based on their primary role as a clinician, government policy maker, health insurer/healthcare organization representative, MBSR instructor, or patient/MBSR student, with the aim to have at least four representatives in each role. A sixth role began to emerge during the interview process, which was healthcare administrator. Advisory board members self-identified their primary and secondary roles. Consistent with previous work (Sekayi & Kennedy, 2017), we selected advisory board members for the perceived expertise they could contribute to the topic of MBSR and its widespread dissemination and implementation. To be included as a participant on the advisory board, members had to: (a) have some degree of administrative, policymaking, or clinical influence that may contribute to our understanding of barriers and facilitators to the widespread use of MBSR; (b) have specific expertise with MBSR as an MBSR teacher; and/or (c) be someone who took MBSR who also has a medical condition. Recruitment initially took place through purposive sampling with consideration of diversity in terms of age, race, gender, and geographical location through established contacts with the investigative team and their colleagues. Secondary recruitment included snowball sampling via professional and social networks and recruitment through professional organization member lists (e.g., Global Mindfulness Collaborative).

### Procedure

We sought participants’ input on three questions about MBSR, aligning with our aims: (1) What should inform if, when, and how MBSR is covered by health insurance? (2) What are the barriers and facilitators to MBSR being covered by health insurance? (3) What should be included in an MBSR systematic review protocol to inform evidence-guided policy-making on health insurance coverage?

Four rounds of participant assessments were conducted in the modified Delphi process. Findings from each round sequentially built the voting process while ensuring representation, clarifications of misunderstandings, opportunities for new items to be introduced where appropriate, and three rounds of member voting, thereby maximizing the likelihood of participant inclusivity and accuracy of ratings.

Before conducting the modified Delphi (Round 0), we used formative evaluation methods (Stetler et al., 2006) to identify participants’ initial recommendations on the questions posed above. As described below, items for the modified Delphi were extracted from qualitative interviews. We then conducted a four-round modified Delphi process (Helmer, 1967), including (a) an initial rating round; (b) a discussion and re-rating round; (c) a final rating round; and (d) a member checking round. This process yielded a list of items related to each aim on which participants achieved consensus based on an a priori threshold (i.e., 80%), as well as a ranked list of all items by mean. A three-round Delphi is generally considered adequate for participants to achieve consensus (Naisola-Ruiter, 2022; Skulmoski et al., 2007). We added an additional member checking round (Birt et al., 2016) (round 4) to reduce bias, ensure that advisory board members agreed with the findings, and elicit any additional feedback on the results and the proposed systematic review protocol based on these results. Items that were rated most highly for consideration (Table 2) used an 80% threshold for agreement, which has been used in a previous work (Connell et al., 2019; Eubank et al., 2016). Specifically, items that were rated with a score indicating that they should probably or definitely be included as a recommendation (i.e., a score ≥ 7 on a 9-point scale) by at least 80% of participants were considered to have met group consensus.

### Measures and Data Analyses

#### Qualitative Interviews (Round 0)

Between September 2022 and January 2023, we conducted individual semi-structured qualitative interviews with Advisory Board members; interviews were informed by the SPIRIT (Supporting Policy in health with Research: An Intervention Trial) Action Framework. The SPIRIT Action framework considers (a) catalysts for research evidence use, (b) capacity for using research, (c) engagement with research, and (d) outcomes that are most critical for this research engagement. The qualitative interview guide featured questions related to each of these domains as they relate to use of evidence about MBSR specifically. Interviews were analyzed using a rapid framework analysis approach in which we created a summary template to distill key points from each interview and transferred those summaries into a matrix (Gale et al., 2019; Hamilton, 2013). The matrix allowed us to view all responses for each interview question in a condensed format. To identify the items that were rated in the modified Delphi, we specifically looked at responses to questions about Aim 1 (if, when, and how MBSR should be covered), Aim 2 (barriers and facilitators to MBSR having its own health insurance billing code and being covered by health insurance), and Aim 3 (beliefs about the highest priority evidence needed on MBSR’s effectiveness and cost-effectiveness). The first author (HEF) used the data from the framework matrix to generate an exhaustive list of items that corresponded to each study aim. Similar statements were grouped together and reworded as needed to create representative responses. The second author (AA) reviewed this list to ensure that it was representative of the qualitative data, to clarify wording, and to further combine any similar items to reduce redundancy. These items were then ranked during the modified Delphi rounds to achieve consensus about the relevant items at play for each aim (Sekayi & Kennedy, 2017). These items (*n* = 149) appear in Table 2 and in Supplementary Information.

#### *Delphi Round 1: Initial Rating *via* Asynchronous Survey*

Round 1 of the modified Delphi took place in January 2023 and included completion of an initial asynchronous survey via Qualtrics in which participants (*N* = 25) rated all items derived from the qualitative interviews. Participants identified whether items for each aim should be included using a rating scale ranging from 1 (*definitely no*) to 9 (*definitely yes*) with the midpoint rating of a 5 representing a response of *uncertain/don’t know*. Question A asked “Should [item] inform if, when and how MBSR is covered by health insurance?” Question B asked “Should [item] be included in the systematic review protocol?” Question C was divided into two parts, which asked “Is [item] a barrier / something that makes it harder for MBSR to be covered by health insurance (e.g., with its own health insurance billing code)?” and “Is [item] a facilitator / something that makes it easier for MBSR to be covered by health insurance (e.g., with its own health insurance billing code)?” In addition to rating the importance of each item, participants also completed a demographics form and rated their knowledge of mindfulness.

#### *Delphi Round 2: Synchronous Discussion and Re-rating *via* Asynchronous Survey*

Round 2 of the modified Delphi took place via Zoom meetings with participants to foster discussion, as well as re-rating of all items after the group meetings via asynchronous survey. All items rated in this survey are represented in Table 2 and in Supplementary Information. To aid scheduling, Zoom meetings were split across two groups. Group 1 (*n* = 10) took place in January 2023 and Group 2 (*n* = 11) took place in February 2023. In the initial meeting for each group, participants were oriented to the modified Delphi process. They were also provided with a description of how items that they rated were selected (i.e., based on qualitative interviews). Then, we provided an initial overview of items to give a summary of how others in their group replied, including (a) items that received high scores on the Round 1 survey (i.e., ≥ 80% of scores ≥ 7); (b) items that received low scores on the Round 1 survey (i.e., ≥ 80 of scores ≤ 3; no items met this threshold); (c) proposed new items by survey respondents; (d) items that were unclear or would benefit from being reworded based on feedback from respondents; (e) items that had high levels of uncertainty (i.e., ≥ 80% of scores in the 3–7 range); and (f) items that elicited the highest level of disagreement (i.e., at least 25% of responses ≤ 4 and at least 25% ≥ 8). Consistent with the approach described by Connell et al. (2019), we focused our discussion time on items that elicited high levels of uncertainty.

We planned to also discuss items with a high level of disagreement, but there was only one item (“financial costs of MBSR to participants,” Aim 1) that met the disagreement criteria. Thus, given time constraints and more items than we would be able to discuss in the 2-hr meeting, we focused on uncertainty items; we conducted a poll via Zoom so that participants could select the top 3 “uncertainty” items that they wanted to discuss for each question. All unclear and new items were discussed for each aim. For each item that was discussed, participants were encouraged to consider how they defined the item, how important it is, and whether the item should be retained, modified, or removed from the final list. Participants were able to share their thoughts verbally or via Zoom chat. Although each group specifically discussed responses to the survey from their group, we consolidated findings across groups when modifying the survey for re-rating. Consistent with previous work (Eubank et al., 2016), statements that did not meet 80% agreement in the initial survey were modified based on feedback from the group discussions prior to redistribution of the survey.

The asynchronous survey was re-administered and completed by participants (*n* = 21) after the synchronous meetings in February 2023. Participants were told to consider information discussed in the group meetings to inform their updated ratings. This survey asked participants to rate *all* items, including those that met 80% agreement in the initial survey to give participants an opportunity to adjust their responses based on what emerged from the group discussion. The means reported in Table 2 are primarily based on responses to this survey. 

#### *Delphi Round 3: Final Rating *via* Asynchronous Survey*

After completion of the Round 2 survey, we reviewed responses and identified items that met the 80% cutoff (i.e., ≥ 80% of scores that were rated ≥ 7). Rather than re-rating those items (since there was already evidence of consensus that they should be included in the final consensus list of items), we asked participants to identify (yes or no) whether those items should be retained. If respondents said “no,” they were asked to provide a rationale. All remaining items were re-rated, including additional items that were added based on participant suggestions from the Round 2 survey (see “Results” for details on the participant suggested items). The final asynchronous survey was administered in April 2023 and completed by 25 participants.

#### Delphi Round 4: Member Checking

After all surveys were completed, we scheduled a synchronous Zoom meeting with advisory board members who responded with their availability (*n* = 14) in April 2023 to present and discuss final results from the survey. For items that were identified as being unclear in the final survey, we asked advisory board members to weigh in on potential wording changes and sought their final approval on items that met the threshold for inclusion. In addition, we presented the final systematic review protocol for member feedback, which was developed primarily based on the items selected for aim 3.

## Results

Demographic characteristics of the study sample are shown in Table 1. Participants' roles included clinicians (*n* = 4), government policymakers (*n* = 6), health insurer and healthcare organization representatives (*n* = 4), MBSR instructors (*n* = 4), patients/MBSR students (*n* = 5), and healthcare administrators (*n* = 3). Following recruitment, participants were invited to endorse any additional roles that applied to them, resulting in the following sample sizes for participants with each domain of expertise: clinicians (*n* = 10), government policymakers (*n* = 7), health insurers and healthcare organization representatives (*n* = 4), MBSR instructors (*n* = 6), patients/MBSR students (*n* = 11), and healthcare administrators (*n* = 4). Race/ethnicity was predominantly White non-Hispanic (77%) followed by Hispanic (12%), Black non-Hispanic (8%), and mixed race (4%). There were similar representations of males and females. There was reasonable geographic representation across the United States, with 23% of participants in the mid-Atlantic, 8% in the Midwest, 8% in the Southeast, 8% in the Southwest, and 12% in the West, recognizing the largest proportion (42%) were in the Northeast. Education levels reflected the jobs of participants, with 73% of participants having a graduate degree. Participants’ self-rated expertise on the study aims was rated on a scale from 0 to 10 (0 = *absolutely no knowledge on the topic*, 10 =* renowned expert on the topic*). Mean scores were consistent with “moderate knowledge” (*M* = 4.75, *SD* = 2.56) with scores ranging from 0 to 9.5. Brief findings from each modified Delphi round are shown below and detailed in Supplementary Information. Overall results follow the description of the round-specific findings.

**Table 1:** Participant demographics

Demographic characteristics	Mean or *n*	*SD* or %
**Age**	46.9	13.5
**Advisory board member primary role**		
Clinician	4	15.4%
Government policy maker	6	23.1%
Healthcare administrator	3	11.5%
Health insurer and healthcare organization representatives	4	15.4%
MBSR instructor	4	15.4%
Patient/MBSR student	5	19.2%
**Race/ethnicity**		
Black, non-Hispanic	2	7.7%
Hispanic	3	11.5%
Mixed race, non-Hispanic	1	3.8%
White, non-Hispanic	20	76.9%
**Gender**		
Female	15	57.7%
Male	11	42.3%
**Geographic region (live and work)**		
Mid-Atlantic	6	23.1%
Midwest	2	7.7%
Northeast	11	42.3%
Southeast	2	7.7%
Southwest	2	7.7%
West	3	11.5%
**Education level**		
College degree	4	15.4%
Graduate degree	19	73.1%
Student	1	3.8%
Data missing	2	7.7%
**Employment status**		
Working full-time	21	80.8%
Working part-time	2	7.7%
Other: full-time student	1	3.8%
Data missing	2	7.7%

### Qualitative Interviews (Round 0) Results

As described above, qualitative interviews were analyzed using a framework-guided rapid analysis approach (Gale et al., 2019; Hamilton, 2013). This resulted in an initial list of items for participants to rate in subsequent rounds of the Delphi process. These items appear in Table 2 and Supplementary Information.

**Table 2:** Items that met Delphi cutoff (i.e., ≥ 80% of participants rated as 7 or higher)

**Aim 1: Should [item] inform if, when and how MBSR is covered by health insurance**	
**Mean** **(*SD*)**	**Items meeting 80% cutoff in round 3 (12.2% of initial items)**
8.48 (0.75)	Research support demonstrating that MBSR works
7.81 (1.23)	MBSR not being harmful/having few side effects for people for whom it is appropriate
7.71 (1.42)	The extent to which MBSR can be clearly defined
7.52 (1.47)	MBSR’s impact on health outcomes as demonstrated by standardized measures
7.48 (1.21)	MBSR teacher qualifications
**Aim 2a: Is [item] a barrier/something that makes it harder for MBSR to be covered by health insurance?**	
**Mean** **(*SD*)**	**Items meeting 80% cutoff in round 3 (10.3% of initial items)**
7.24 (1.42)	Perception that MBSR is not a medical treatment*
7.04 (1.40)	Patient barriers to attending an 8-week MBSR course
7.00 (1.50)	Challenges with the potential delivery of a health service by a non-licensed provider (or need to develop a licensing process)
**Aim 2b: Is [item] a facilitator/something that makes it easier for MBSR to be covered by health insurance?**	
**Mean (*SD*)**	**Items meeting 80% cutoff in Round 3 (41.2% of initial items)**
8.19 (0.93)	MBSR’s potential for addressing common problems like depression and anxiety
8.05 (1.32)	Evidence that MBSR is effective at treating mental health and psychosomatic problems
8.00 (1.10)	The ability to deliver MBSR via telehealth
8.00 (1.14)	Buy-in of large professional groups providing this service (e.g., psychology and social work state or national groups)
7.95 (1.56)	MBSR being established as an intervention that works
7.95 (0.97)	Partnerships with reputable institutions to demonstrate effectiveness
7.86 (1.06)	The ability to demonstrate that MBSR is being delivered with fidelity (i.e., teachers deliver its core components)
7.76 (1.04)	National interest in evidence-based approaches that improve mental health outcomes
7.71 (1.19)	Positive public perceptions of mindfulness/MBSR
7.67 (1.24)	MBSR’s standardized curriculum that can be used anywhere (i.e., replicability and scalability)
7.67 (1.39)	MBSR’s ability to address stress broadly
7.57 (1.57)	Potentially lower out of pocket costs for MBSR than for therapy
7.48 (1.12)	Increased acknowledgement by insurers about the value of mindfulness-based interventions
7.44 (1.56)	Cost-effectiveness of MBSR
7.24 (2.10)	Minimal side effects of MBSR
**Aim 3: Should [item] be included in the systematic review protocol?**	
**Mean** **(*SD*)**	**Items meeting 80% cutoff in round 3 (44.2% of initial items)**
8.29 (1.19)	Conditions that are effectively treated with MBSR
8.10 (1.22)	MBSR’s impact on stress
8.00 (1.30)	MBSR’s impact on PTSD/trauma symptoms
7.95 (1.24)	MBSR’s impact on pain catastrophizing
7.90 (1.18)	MBSR’s impact on pain symptoms
7.86 (1.32)	MBSR’s impact on anxiety
7.86 (1.20)	MBSR’s impact on chronic illness symptoms
7.81 (1.25)	MBSR’s impact on quality of life
7.76 (1.26)	MBSR’s impact on cardiometabolic risk
7.68 (1.55)	How MBSR compares to other interventions (comparative effectiveness)
7.67 (1.68)	Trajectory of symptom improvement following MBSR
7.67 (1.28)	Dose of MBSR (i.e., class length, home practice)
7.67 (1.20)	Mechanisms of MBSR (i.e., how and why it works)
7.64 (1.35)	Costs and potential savings (to insurance companies) associated with MBSR
7.62(1.32)	Long-term outcomes of MBSR (e.g., effect on quality of life)
7.62 (1.28)	MBSR’s impact on depression symptoms
7.52 (1.08)	Extent to which MBSR reduces service utilization (e.g., hospital readmissions, avoidable emergency department use, doctor’s appointments)
7.52 (1.83)	Comparisons between MBSR and other mindfulness-based interventions
7.48 (1.33)	How MBSR works in combination with other treatments (e.g., medication, therapy)

All items met the 80% cut off in Round 2 and means reflect ratings in the Round 2 survey. These items were then confirmed as being retained for inclusion via a dichotomous yes/no response in Round 3; all items that met the cut off in Round 2 were also endorsed for inclusion in Round 3. Items with an asterisk (*) were added in Round 2

### Round 1 (Asynchronous Survey) Results

Participants who completed the initial survey (*n* = 25) generated eight potential new items (e.g., “Perception that MBSR is not a medical treatment” for Aim 2a) and further wording clarifications that were discussed in Round 2 for potential inclusion in future iterations of the survey. The specific items are shown in Supplementary Information.

### Round 2 (Synchronous Discussion and Re-rating via Asynchronous Survey) Results

Following the synchronous discussion with participants after the initial survey, six of the eight newly generated items were selected for inclusion on the next survey. Based on discussion, several wording changes were made to the newly proposed items, resulting in several new items, detailed in Supplementary Information. Examples of new items include (a) coverage policies of peer payers (*Question 1*) and (b) the potential for MBSR to be covered via mechanisms other than fee-for-service, such as value-based contracts, capitation, and bundled services (*Question 1*). In addition, some existing items were reworded based on group feedback and discussion, as described in Supplementary Information.

The asynchronous survey that was administered following group discussion included the edits described above. Items that met the 80% threshold (i.e., at least 80% of participants rated an item as ≥ 7) are shown in Table 2 along with their mean scores. These represent the items that advisory board members agreed are most critical to address to advance health insurance coverage for MBSR.

### Round 3: Final rating via Asynchronous Survey Results

As described above, the Round 3 survey provided participants with an opportunity to confirm that they agreed with the items that met the 80% threshold (i.e., at least 80% of participants rated an item as ≥ 7) for inclusion. All items were retained for final inclusion. No new items were identified in the Round 3 survey. Items that did not meet the 80% cutoff, but were rated in the Round 3 survey, are shown in Supplementary Information.

### Round 4: Member Checking Results

During the member checking meeting, participants were provided with an opportunity to suggest final changes and to discuss points for clarification. Overall, participants agreed with the big-picture results of the survey. They suggested wording changes for three of the items that were selected for final inclusion (i.e., that met the 80% threshold), detailed in Supplementary Information.

Another key component of the member checking meeting was to garner feedback on the most important topics for MBSR evidence syntheses, including systematic reviews and meta-analyses, informed primarily by the highly rated items from Aim 3. Based on a review of items selected for final inclusion, the most highly rated systematic review topics were presented to the advisory board. To organize this presentation, the investigator leading the systematic review (BTJ) described these highly rated items as falling into three topics, including (a) risk–benefit ratio of MBSR; (b) scalability/accessibility of MBSR; and (c) technical components and resources related to MBSR delivery. Advisory board members were provided with the opportunity to discuss questions, concerns, and proposed adjustments to the evidence synthesis priorities. Several points emerged through this discussion, including the importance of assessing “improvement” following MBSR in terms of both reduced symptoms and improved quality of life, the need to assess whether MBSR can be effectively delivered via telehealth, and considerations related to equity such as whether MBSR outcomes are comparable across diverse racial and ethnic groups and delivery in different languages. Further findings relevant to recommendations for the systematic review appear in Supplementary Information.

### Overall Findings

The overall findings following all rounds of participant assessments, with an emphasis on rank and scoring the importance of the recommendations, appear in Table 2. Out of the initial 149 items that were ranked, 42 (28.2%) were retained for inclusion across all aims. The top three highest items that should inform if, when, and how MBSR is covered by health insurance (i.e., Aim 1) were (a) research support demonstrating that MBSR works (*M* score = 8.48); (b) MBSR not being harmful/having few side effects for people for whom it is appropriate (*M* score = 7.81); and (c) the extent to which MBSR can be clearly defined (*M* score = 7.71).

The top three items that were considered a barrier, or something that makes it harder for MBSR to be covered by health insurance (i.e., Aim 2a), were (a) the perception that MBSR is not a medical treatment (*M* score = 7.24); (b) patient barriers to attending an 8-week MBSR course (*M* score = 7.04); and (c) challenges with the potential delivery of a health service by a non-licensed provider (or need to develop a licensing process; *M* score = 7.00).

The top three items that were considered a facilitator, or something that makes it easier for MBSR to be covered by health insurance (i.e., Aim 2b), were (a) MBSR’s potential for addressing common problems like depression and anxiety (*M* score = 8.19); (b) evidence that MBSR is effective at treating mental health and psychosomatic problems (*M* score = 8.05); and (c) the ability to deliver MBSR via telehealth (*M* score = 8.00), and (tied for third place), buy-in of large professional groups providing this service (e.g., psychology and social work state or national groups; *M* score = 8.00).

Finally, the top three items related to the highest priority evidence needed to inform health insurance coverage decisions (Aim 3) were (a) conditions that are effectively treated with MBSR (*M* score = 8.29); (b) MBSR’s impact on stress (*M* score = 8.10); and (c) MBSR’s impact on PTSD/trauma symptoms (*M* score = 8.00). Other items listed in order of average score are detailed in Table 2.

As a closing activity during the final member checking meeting, advisory board members shared their hopes for MBSR in the next 5 years. These included attaining a billing code that would allow for MBSR to be covered by insurance as a standalone intervention, moving MBSR into “mainstream” healthcare and leveraging its potential to transform how we think about health, integrating it into a curriculum for younger audiences (e.g., pediatric behavioral health), and building greater leadership buy-in and practice of mindfulness.

Participants were also asked to generate ideas for dissemination strategies for findings about MBSR. These included broad sharing of public service announcements, targeted messages/summaries of findings about MBSR, making mindfulness part of routine patient care and thus requiring provider training, increasing the number of MBSR providers who are within network, and networking with healthcare systems and global collaboratives to share information about research findings related to MBSR.

## Discussion

This study used key informant evaluations and a modified Delphi method with participants who formed an advisory board of health insurers, healthcare administrators, policymakers, clinicians, MBSR instructors, and MBSR students. Using an iKT approach, the study team collaborated with the advisory board to co-create knowledge that will inform future research and policy related to MBSR. Items selected for final inclusion from the Delphi rounds represent five priorities to consider in advancing MBSR health insurance coverage. These priorities include (a) addressing the health impacts of MBSR, (b) considering the patient experience, (c) understanding costs, (d) assessing teacher fidelity to MBSR, and (e) the value of involvement with professional organizations. Notably, the first three priorities align with the policy-relevant triple aim (Berwick et al., 2008) of improving health, improving individuals’ experiences of care, and reducing costs of care*.*

With regard to the first mentioned triple aim of *improving health impacts*, participants endorsed the importance of research support demonstrating that MBSR works on numerous health and mental health outcomes (e.g., stress, PTSD, pain, anxiety, chronic illness symptoms, quality of life, cardiometabolic risk). Individual qualitative interviews (Round 0 of the Delphi) were informed by the SPIRIT Action Framework, which considers capacity for, engagement with, and use of research findings. Research support for MBSR was consistently endorsed as a priority by advisory board members who varied in the extent to which they use research regularly. This underscores general interest in evidence-based interventions, though many participants named that they do not always know whether treatments—including MBSR—are backed by research. Thus, more work is needed to provide timely and digestible information to key informants about research findings in a tailored way (e.g., via dissemination strategies (Purtle et al., 2020)).

The second priority of *patient experience* resulted in several recommendations related to dose, delivery, and access. Standardized MBSR involves 2.5-hr sessions once a week for 8 weeks, as well as a 7.5-hr retreat. Although this version of MBSR is the one that has most been empirically tested, such a high “dose” of the intervention may not be feasible for all patients. Thus, there are questions about the need to maintain fidelity to standardized MBSR while balancing pragmatic considerations for its widespread delivery. One participant—a health insurer—mentioned how sizable efforts can be made by health insurers to bill for behavioral interventions, but if people do not attend the interventions, the efforts are meaningless. At a time when there are many types of mindfulness training available, the market strength for MBSR should continue to be monitored. Enrollments for MBSR in the United States and in many global regions continue to be modestly strong and would likely increase if costs were reduced through health insurance coverage. MBSR can be delivered in lower doses, but it may no longer be appropriate to name these iterations “MBSR” (Crane et al., 2017; Loucks et al., 2022). Efforts to identify the “core components” (Damschroder et al., 2009; Fixsen et al., 2005) that are essential to MBSR will inform future iterations of the intervention that maintain its integrity while also addressing patient-level barriers to attendance. For instance, delivery modality (i.e., online versus in person) seems likely within the “adaptable periphery” (Damschroder et al., 2009; Fixsen et al., 2005) of MBSR. Changing delivering modality to online in some instances may make attendance easier. Consistent with this, participants rated “the ability to deliver MBSR via telehealth” as an important facilitator to its widespread implementation. Future work should further examine opportunities for adapting MBSR to address patient needs.

The third element of the triple aim, *reducing costs of care*, is important and under-researched. Whether costs are accrued by the individual user (patient) or by the government, costs influence decisions about what is affordable and highest priority to cover. Findings showed high rankings of elements of cost-effectiveness, such as costs, potential savings, and the extent to which MBSR reduces service utilization. Previous studies have examined MBSR cost-effectiveness (e.g., D’Amico et al., 2024; Feliu-Soler et al., 2018; Herman et al., 2017; Knight et al., 2015; Lengacher et al., 2015; Perez-Aranda et al., 2019), but methodological quality of these studies has been variable. A recent systematic review provides promising evidence that MBSR is cost-effective across nine countries, including the United States, even reducing costs to both payers and society (Zhang et al., 2022). There are impactful opportunities to replicate and extend cost-effectiveness findings on MBSR. Indeed, we saw in the current study that cost-effectiveness was highly rated as a facilitator that makes it easier for MBSR to be covered by health insurance, including “potentially lower out of pocket costs for MBSR than for therapy” and “cost-effectiveness of MBSR.”

The fourth priority, *teacher fidelity to the research-based program*, was reflected in highly rated items (e.g., “MBSR teacher qualifications”) and was a frequent topic of discussion among key informants. There are efforts in the United States and worldwide to provide high-quality MBSR teacher training programs, including with organizations such as The Global Mindfulness Collaborative and the British Association of Mindfulness-based Approaches (BAMBA), alongside specific university-affiliated programs such as through Brown University’s School of Professional Studies, the University of California San Diego’s Center for Mindfulness, Bangor University in Wales, and Aarhus University in Denmark. These organizations provide standardized credentialing programs to ensure high-quality MBSR teacher training and offer the capacity to scale up teacher training as needed if shifts in health insurance coverage increase access and demand. The “Mindfulness-Based Interventions: Teaching Assessment Criteria” (MBI:TAC) tool was developed to foster high-quality teacher training with qualitative and quantitative feedback to trainees on skills in domains identified to be important for mindfulness teachers, including (a) coverage, pacing, and organization of session curriculum; (b) relational skills; (c) embodiment of mindfulness; (d) guiding mindfulness practices; (e) conveying course themes through interactive inquiry and didactic teaching; and (f) holding the group learning environment (Griffith et al., 2021). Several MBSR teacher-training programs use these criteria to guide teacher development and evaluate trainees for MBSR teacher certification.

Finally, the importance of professional organizations was highlighted. For example, “buy-in of large professional groups providing this service (e.g., psychology and social work state or national groups)” was rated highly (Table 2). This finding gave us pause, as we are aware of almost no statements by professional groups recommending MBSR, such as the American Medical Association or the American Public Health Association. The closest examples we found were (a) the U.S. Department of Veterans Affairs Evidence Synthesis Program, which requested the U.S. Department of Veterans Affairs Evidence Synthesis Program to conduct a systematic review of MBSR trials (Hempel et al., 2014); (b) the Agency for Healthcare Research and Quality (AHRQ) which contracted a systematic review on meditation programs that included MBSR in 2014 (Goyal et al., 2014a, 2014b); (c) the 2019 *Pain Management Best Practices Inter-Agency Task Force Report* by the United Sates Department of Health and Human Services, which described MBSR as a recommended approach to manage pain (United States Department of Health & Human Services, 2019, May); (d) a statement on meditation and cardiovascular risk reduction from the American Heart Association (Levine et al., 2017); (e) a statement from the American College of Physicians to include nonpharmacological approaches to pain management, including MBSR (Qaseem et al., 2017), though this has not yet resulted in coverage changes (Bonakdar et al., 2019). Based on feedback from key informants in this study, efforts to partner with national and international professional organizations who are willing to endorse MBSR have the potential to advance health insurance coverage. This will require coordinated efforts between researchers and policymakers to ensure that recommendations are indeed translated into practice.

Notably, other mindfulness-based and mindfulness-informed programs have been assessed and recommended by professional organizations. The National Institute for Healthcare Excellence (NICE) in the UK recommends Mindfulness-Based Cognitive Therapy (MBCT) for prevention of depression relapse and as a treatment for less severe depression (National Institute for Health & Care Excellence, 2022). Thus, MBCT has health insurance coverage in the UK. Other fields, such as counseling, provide the example of “sunrise laws” in which a profession seeking licensure proposes elements of legislation, alongside the costs and benefits, and works with governing bodies to pass legislation (Bergman, 2013). There are opportunities for MBSR teachers and teacher training organizations to collaborate to provide a uniform voice and strategy for public and private acceptance, alongside licensure criteria that would create a uniform standard for MBSR teachers in every state. In the process of recruiting policymakers to the study, we spoke with several staff members of policymakers who were willing to have informal conversations about the topic. One shared that before she would bring MBSR health insurance coverage to “her boss” (a U.S. senator) to consider advocating for health insurance coverage, she would want to know what professional organizations recommended. Policymakers rely on the recommendations of respected health organizations to support them in advocating for evidence-informed policy changes. There are opportunities for professional organization writing groups to provide evidence-based statements on the impacts of MBSR on health outcomes so that policymakers can point to these when considering policy shifts in health insurance coverage of MBSR. From this study support such efforts to write consensus statements from professional organizations, and we encourage the field to do so.

### Limitations and Future Research

This study has multiple strengths including its use of iKT methods, the diversity of key informant perspectives, and use of the modified Delphi approach to systematically achieve consensus. The study also has limitations. First, certain participants in the Delphi discussion may have been less comfortable sharing their perspectives during group discussions and their thoughts may be less represented. Although efforts were made to encourage all participants to share their thoughts (e.g., option to speak verbally or use group chat anonymously; calling on specific subgroups for their perspectives on certain questions), people representing the MBSR student perspective spoke least frequently during the Delphi discussions. Scholars studying focus groups have recognized that there is an inevitable influence of group dynamics on individual responses, which can result in minimizing or withholding experiences (Cyr, 2016). This limitation was countered by conducting one-on-one qualitative interviews with all advisory board members in Round 0 to gather their recommendations. Furthermore, each member had an equal and anonymous vote to rate the importance of each item, such that participants who spoke less still had the same weighting of their vote as those who spoke more.

Second, although we made efforts to recruit a diverse sample in terms of race/ethnicity and geographic region, increased diversity in these areas may have yielded somewhat different results. Limited racial/ethnic diversity of our sample may also reflect a larger systemic problem in which people from racial and ethnic minoritized backgrounds are underrepresented in many of the roles sought for this study (e.g., policymakers, health insurers). In addition to systemic change, more MBSR research that centers the perspectives of minoritized groups is needed. Existing work with minoritized populations highlights the importance of emphasizing cultural-centric teachings (e.g., Afro-centric, Native American-centric), training culturally concordant MBSR teachers, and leveraging natural strength in these communities, such as ties to family and community (Crane et al., 2023; Nagy et al., 2022; Proulx et al., 2018; Tenfelde et al., 2018; Woods-Giscombé & Gaylord, 2014).

Third, the advisory board represents a group of people who were sufficiently interested in MBSR to be willing to participate in ongoing meetings and surveys; there may be some opinions that are not represented by people unwilling to attend meetings on this topic. Similarly, a larger number of experts or a different group may have led to different results. This limitation is mitigated by the fact that we used a rigorous recruitment strategy and set a high pre-established criterion for agreement to reach consensus on items selected for final inclusion.

A final limitation is that the sample size was not adequate to do stratified statistical analyses by key informant type. Certain topics may be more important to some key informant roles (e.g., policymakers, health insurers). Our results show ranking of importance across the range of key informants; future research could compare responses within specific informant groups to better understand drivers for each group type.

This study provides an overview of priority considerations for advancing health insurance coverage for MBSR. We are at a time in history with strong public interest in mindfulness, a building evidence base including for MBSR, and inequities in access to evidence-based mindfulness programs that have gone through extensive randomized controlled trial research. Health insurance coverage of evidence-based mindfulness programs would improve inequities in access to evidence-based care by reducing the economic burden to those for whom it is difficult to pay. This study’s findings suggest several areas that would influence MBSR health insurance coverage, including health impacts, patient experience, costs, teacher fidelity to the research-based program, and the value of involvement with professional organizations. These five areas are recommended priorities when working to advance MBSR health insurance coverage.

### Supplementary Information

Below is the link to the electronic supplementary material.

Supplementary file1 (DOCX 34 KB)

## Data Availability

De-identified data from this study are not available in a public archive. De-identified data from this study may be made available upon request. Requests may be sent to the corresponding author.

## Declarations

### Use of Artificial Intelligence

Artificial intelligence was not used in the preparation of this manuscript.
