# Supplementary material for: Mindfulness-Based Stress Reduction Health Insurance Coverage: If, How, and When? An Integrated Knowledge Translation (iKT) Delphi Key Informant Analysis
Source: Mindfulness (N Y). 2024 May 17;15(5):1220–33. doi: 10.1007/s12671-024-02366-x (PMC11133142; doi:10.1007/s12671-024-02366-x)
Supplement: Supplementary file 1 — Supplementary file1 (DOCX 34 KB) [file 12671_2024_2366_MOESM1_ESM.docx]

**Supplementary Information**

**Detailed Results from Participant Interviews and Modified Delphi Approach**

**Qualitative Interviews (“Round 0”)**

Responses from qualitative interviews were used to generate an exhaustive list of items for participants to rate in subsequent rounds of the Delphi process. These items are represented in Tables 2 and 3 with the exception of items marked with an asterisk, which were generated in Round 2 as described below. In-depth results from qualitative interviews will be reported elsewhere.

**Round 1 (Asynchronous Survey)**

Participants who completed the initial survey (*N*=25) generated several potential new items (*N*=8) and wording clarifications that were discussed in Round 2 for potential inclusion in future iterations of the survey. These proposed new items included: (1) Coverage policies of peer payers (*Aim 1*); (2) Feasibility of the traditional MBSR format versus a shorter intervention (*Aim 1*); (3) If MBSR is something an employer would want to pay for and have in their benefits package (*Aim 1*); (4) Coverage on a fee-for-service basis may not be the only way to achieve payer support of MBSR (*Aim 1*); (5) Determining the volume of MBSR providers that would meet the needs of a population (*Aim 2a: Barriers*); (6) The closeness of MBSR to other approved therapies (*Aim 2a: Barriers*); (7) bureaucratic inertia (*Aim 2a: Barriers*); and (8) MBSR’s ease of use is a benefit to patients (*Aim 2a: Barriers*).

**Round 2 (Synchronous Discussion and Re-Rating via Asynchronous Survey)**

Following the synchronous discussion with participants after the initial survey, six of the eight newly generated items were selected for inclusion on the next survey. Based on discussion, several wording changes were made to the newly proposed items, resulting in the following new items: (1) Coverage policies of peer payers (*Aim 1)*; (2) The potential for MBSR to be covered via mechanisms other than fee-for-service, such as value-based contracts, capitation, bundled services (*Aim 1*); (3) Perception that MBSR is not a medical treatment (*Aim 2a: Barriers*); (4) Accessing MBSR requires financial privilege, including work flexibility (*Aim 2a: Barriers*); (5) The complex and time-consuming process to navigate relevant systems (e.g., CPT code system, Medicaid, legislation process, other bureaucracy) (*Aim 2a: Barriers*); (6) MBSR’s ease of use is a benefit to patients (*Aim 2b: Facilitators*). In addition, the following existing items were reworded based on group feedback and discussion with italics indicating wording additions: (1) MBSR’s impact on health outcomes *as demonstrated by standardized measures*; (2) What MBSR provider network would look like (e.g., how many providers? *Who is qualified to teach*?) (3) *Variability in* financial costs to MBSR participants *(based on factors like health insurance and other resources)*; (4) Evidence that MBSR is widely performed nationally (*i.e., are there providers in most geographical areas?*); (5) Availability of MBSR services *either locally or via telehealth.*

The asynchronous survey that was administered following group discussion included the edits described above. Items that met the 80% threshold (i.e., at least 80% of participants rated an item as ≥7) are shown in Table 2 along with their mean scores. These represent the items that advisory board members agreed are most critical to address to advance health insurance coverage for MBSR.

**Round 3: Final rating via Asynchronous**

As described above, the Round 3 survey provided participants with an opportunity to confirm that they agreed with the items that met the 80% threshold for inclusion. All items were retained for final inclusion. No new items were identified in the Round 3 survey. Items that did not meet the 80% cut off but that were rated in the Round 3 survey are shown in Table 3 alongside their mean scores.

**Round 4: Member Checking**

During the member checking meeting, participants were provided with an opportunity to suggest final changes and to discuss points for clarification. Overall, participants agreed with the big-picture results of the survey. They suggested wording changes for three of the items that were selected for final inclusion (i.e., that met the 80% threshold). Wording deletions are indicated with strikethroughs and additions are indicated in italics: (1) MBSR not being harmful/having few side effects *for people for whom it is appropriate*; (2) MBSR being ~~well-~~established *as an intervention that works for people*; (3) National interest in *evidence-based* approaches that improve mental health outcomes.

Three of the items that met the *a priori* cut off were selected by participants for group discussion. First, participants requested to discuss the Aim 1 item, “The extent to which MBSR can be clearly defined.” They noted that this item captures the need to define MBSR in terms of core components and with a standard curriculum that can be replicated. Participants noted the importance of allowing for some differences in the exact way in which the curriculum is carried out (i.e., its adaptable periphery^31^), but that there are essential elements, such as the body scan and attentional focus meditation that are core to MBSR (i.e., core components^32^). Participants also requested to discuss two Aim 2a (Barriers) items. The item, “Challenges with the potential delivery of a health service by a non-licensed provider (or need to develop a licensing process)” brought up a discussion about whether MBSR providers would need to be licensed to receive health insurance coverage. Some participants noted that there is precedent for non-licensed providers (e.g., doulas, peer recovery specialists) to be able to receive insurance reimbursement for their services. Doing so would require identifying clear credentialing standards and requirements for someone to be called an MBSR instructor who is able to receive reimbursement. Finally, participants discussed “Patient barriers to attending an 8-week MBSR course.” They noted the importance of considering whether MBSR is feasible for patients to attend. Some feasibility considerations included: (1) Dose – or ensuring that patients are able to get the full benefit of the treatment: (2) Marketability – or whether people can actually engage in what is being offered; and (3) Equity considerations and a desire to avoid making MBSR a “benefit of privilege” that requires flexibility and resources that may not be available to many patients. Advisory board members identified that there might be value in assessing a lower dose or a different delivery model of MBSR that might reduce patient barriers and increase equity.

Another key component of the member checking meeting was for the systematic review protocol to be presented to the advisory board. The systematic review protocol was informed primarily by the highly rated items from Aim 3. Based on a review of items selected for final inclusion, a systematic review protocol was presented to advisory board and included three categories that emerged from the data collected from participants: (1) Risk-benefit ratio of MBSR; (2) Scalability/accessibility of MBSR; (3) Technical components and resources related to MBSR delivery. Advisory board members were provided with the opportunity to discussion questions, concerns, and proposed adjustments to the protocol. Several points emerged through this discussion, including the importance of assessing “improvement” following MBSR in terms of both reduced symptoms and improved quality of life, the need to assess whether MBSR can be effectively delivered via telehealth, and considerations related to equity such as whether MBSR outcomes are comparable across diverse racial and ethnic groups and delivery in different languages. Some topics were identified by the study team as being more challenging to assess with a systematic review (e.g., cost savings to insurers; public perceptions of mindfulness) but remain important to advisory board members as areas for future research. Advisory board members also underscored the importance of the multiple factors that influence health insurance coverage of MBSR, including decisions made by government policymakers (especially CMS and state Medicaid offices), the role of healthcare professional associations (e.g., American Medical Association, American Public Health Association), and groups such as the United States Preventive Services Task Force. Although some of these topics will not be directly considered with a systematic review, these do highlight priorities for key informants in the pursuit of wider spread health insurance coverage for MBSR.

**Table S1.** Remaining items that did not meet Delphi cut off, ranked by mean score.

| **Aim 1: Should [item] inform if, when and how MBSR is covered by health insurance?** | |
| --- | --- |
| **Mean (SD)** |  |
| 7.36 (1.73) | How MBSR outcomes compare to alternatives (e.g., medication, therapy) |
| 7.24 (1.45) | Whether quality of MBSR can be measured |
| 7.00 (1.38) | Endorsement by professional organizations (e.g., the American Psychological Association) that MBSR is the standard for care |
| 6.96 (1.74) | Cost-benefit analyses (e.g., Is MBSR less expensive than alternative treatments while still leading to comparable outcomes? Can MBSR prevent onset of conditions that are expensive to insurance companies?) |
| 6.92 (1.47) | Who is allowed to teach MBSR (i.e., licensed providers, certified teachers) |
| 6.88 (1.64) | Whether fidelity to MBSR (i.e., teachers delivering its core components) can be measured |
| 6.80 (1.96) | Whether MBSR coverage improves triple aim of quality of care, health outcomes, and cost |
| 6.64 (1.68) | How much MBSR can be distinguished from other mindfulness interventions |
| 6.56 (1.66) | What the MBSR provider network would look like (e.g., how many providers, who is qualified to teach) |
| 6.48 (2.00) | Evidence that MBSR is appropriate for a large part of the population |
| 6.48 (2.06) | MBSR’s return on investment |
| 6.28 (2.01) | MBSR’s cultural appropriateness for a range of populations |
| 6.28 (2.41) | Whether medications are covered by insurance companies to treat a problem that could also be treated with MBSR (e.g., anxiety) |
| 6.24 (2.19) | Financial costs of MBSR to insurance companies |
| 6.20 (1.76) | Feasibility of the traditional MBSR format versus a shorter intervention |
| 6.20 (2.16) | Patients’ willingness to engage with MBSR (i.e., commitment to all classes and activities) |
| 6.16 (1.60) | Whether MBSR can be taken multiple times and still be covered |
| 6.08 (1.98) | Billing structure of MBSR (e.g., by session, by class) |
| 6.04 (2.01) | Availability of MBSR services either locally or via telehealth |
| 6.04 (2.19) | Evidence that MBSR is widely performed nationally (i.e., are there providers in most geographical areas?) |
| 6.00 (2.02) | Patients’ presenting problems/diagnoses |
| 6.00 (1.73) | Potential for MBSR to be covered via mechanisms other than fee for service, such as value-based contracts, capitation, bundled services* |
| 6.00 (2.04) | The benefit classification of MBSR (i.e., medical, behavioral health, preventative) |
| 5.96 (2.05) | The extent to which MBSR can address inequities in care (e.g., provider shortages, waitlists for treatment, access to care) |
| 5.88 (1.97) | Whether MBSR can help people navigate a crisis |
| 5.80 (1.87) | Coverage policies of peer payers (i.e., Even if MBSR has its own billing code, different insurance companies might cover it differently)* |
| 5.72 (2.46) | MBSR’s impact on engagement in routine healthcare screening |
| 5.64 (1.91) | Whether and how other countries cover MBSR |
| 5.60 (1.71) | Patients’ ability to engage with MBSR (e.g., Do they have the necessary resources to make it to classes?) |
| 5.52 (2.29) | Whether MBSR can be taken at the same time as other similar interventions (e.g., therapy) |
| 5.48 (2.02) | If MBSR is something an employer would want to pay for and have in their benefits package |
| 5.48 (2.10) | Variability in financial costs of MBSR to participants (based on factors like health insurance and other resources) |
| 5.24 (2.37) | Anecdotal and testimonial evidence from students that MBSR works |
| 5.16 (2.19) | Patient eligibility for receiving health insurance coverage for MBSR. |
| 5.12 (1.56) | Benefits to providers for being in-network MBSR teachers |
| 3.76 (2.67) | If MBSR treats diseases that Democrats and Republicans are already putting money toward |
| **Aim 2a: Is [item] a barrier / something that makes it harder for MBSR to be covered by health insurance?** | |
| **Mean (SD)** |  |
| 6.96 (1.34) | Misconceptions about MBSR (e.g., how it differs from general mindfulness, that it is a “new wave thing”, that it may conflict with religious beliefs) |
| 6.92 (1.80) | The need to define MBSR and differentiate it from existing services (e.g., group therapy, other mindfulness interventions) |
| 6.76 (1.56) | The complex and time-consuming process to navigate relevant systems (e.g., CPT code system, Medicaid, legislation process, other bureaucracy)* |
| 6.64 (1.44) | Whether alternate interventions (e.g., CBT, pharmacological treatments) have stronger evidence than MBSR to treat an identified problem |
| 6.40 (1.73) | Varying levels of coverage across insurance companies |
| 6.36 (1.47) | The process of changing existing payer reimbursement policies |
| 6.36 (1.78) | A lack of medical provider endorsement |
| 6.36 (1.87) | Insufficient information about most appropriate type of coverage (i.e., clinical, wellness, or preventative service) |
| 6.28 (2.11) | The process of obtaining a billing code |
| 6.28 (1.67) | Cost-effectiveness of MBSR (i.e., whether it is profitable or reduces expenses; if it loses money) |
| 6.28 (1.49) | The need to identify appropriate diagnoses for an MBSR billing code |
| 6.24 (1.48) | Challenges with cost sharing (i.e., Who is going to pay what?) |
| 6.16 (2.04) | Accessing MBSR requires financial privilege including work flexibility* |
| 6.16 (1.99) | Potential for geographic inequity if MBSR classes only take place in person |
| 6.12 (2.24) | Lobbying power of big pharma and insurance companies could hinder approval of MBSR coverage |
| 6.12 (1.88) | Difficulty maintaining the integrity of the MBSR teacher certification process |
| 6.08 (1.58) | Difficulties with reach (i.e., ensuring that the right population can receive and benefit from MBSR) |
| 6.04 (1.65) | Issues with interstate licensing for MBSR instructors |
| 5.96 (1.99) | Marketing of MBSR (term is not well known) |
| 5.96 (2.13) | Difficulty maintaining the integrity and fidelity of MBSR courses |
| 5.92 (1.73) | Challenges with standardizing training |
| 5.92 (2.24) | The lack of standardization of mindfulness tools |
| 5.84 (1.93) | The standardized MBSR curriculum that may not be easily adapted |
| 5.84 (1.52) | Determining the volume of MBSR providers that would meet the needs of a population |
| 5.80 (1.66) | Patient concerns about having the required diagnoses (e.g., mental health diagnoses) for MBSR listed in their chart |
| 5.80 (2.00) | Limited government funding for initiatives that might support MBSR |
| 5.76 (2.26) | The credibility/worthiness of MBSR |
| 5.68 (1.82) | The need for an assessment process to identify if patients have an appropriate diagnosis for MBSR |
| 5.64 (2.41) | Billing infrastructure and support |
| 5.64 (2.41) | Lack of awareness and prioritization of issues like MBSR coverage by congressional members |
| 5.56 (1.42) | Patient compliance and difficulty making up missed MBSR classes |
| 5.44 (1.87) | Costliness of running MBSR |
| 5.44 (2.22) | MBSR may be less appealing to Republicans |
| 5.12 (2.24) | Challenges related to in-person class logistics (e.g., space, equipment) |
| 5.08 (2.02) | The ownership/trademarking of MBSR |
| 4.88 (2.33) | The potential for MBSR to cause worsening of symptoms (e.g., anxiety and other documented adverse effects) |
| **Aim 2b: Is [item] a facilitator / something that makes it easier for MBSR to be covered by health insurance?** | |
| **Mean (SD)** |  |
| 7.40 (1.47) | Potential for MBSR to reduce/replace therapy and/or medication |
| 7.16 (1.21) | Mindfulness being accepted by the government as a valuable intervention |
| 7.12 (1.83) | The transdiagnostic nature of MBSR (i.e., evidence that it works on multiple different conditions) |
| 7.12 (1.62) | Having policymakers who are champions of MBSR |
| 7.08 (1.53) | The delivery of MBSR by licensed clinicians |
| 7.08 (1.63) | MBSR’s approach to normalizing stress responses |
| 7.04 (1.51) | The presence of public champions of MBSR (e.g., policymakers, social media influencers, podcasts) |
| 7.00 (1.61) | The ability of MBSR to benefit an accountable care organization model (groups of doctors, hospitals, and other healthcare providers who come together to give coordinated high-quality care to Medicare patients) |
| 7.00 (1.94) | MBSR’s ease of use is a benefit to patients* |
| 6.88 (1.59) | MBSR being a time-limited intervention with known parameters and costs |
| 6.80 (1.68) | The potential to integrate technology with MBSR (e.g., companion app, online modality) |
| 6.76 (1.92) | Potential for MBSR to fit more easily into providers’ scope of practice than other complementary health approaches (e.g., tai chi, yoga) |
| 6.68 (1.91) | The ability to pilot MBSR with a few medical practices or groups |
| 6.64 (1.96) | Potential for higher acceptability and less stigma related to being an MBSR student compared to being in therapy |
| 6.40 (1.78) | The appeal of MBSR to employers who are buying insurance or self-insured |
| 6.24 (2.19) | Messaging about MBSR that convinces both political parties about its importance and garners bipartisan support |
| 6.16 (2.06) | The potential for MBSR to be offered by a large corporation |
| 6.00 (2.20) | Student testimonials that MBSR works |
| 6.00 (1.96) | Similar benefits to complementary health approaches (e.g., yoga) |
| 5.84 (2.25) | Distributing educational information about MBSR to patients |
| 4.88 (1.99) | MBSR may be more appealing to Democrats |
| **Aim 3: Should [item] be included in the systematic review protocol?** | |
| **Mean (SD)** |  |
| 7.12 (1.88) | Whether completing MBSR reduces therapy/psychotropic medication use |
| 7.08 (1.44) | Feasibility of patient engagement in MBSR, as assessed by attrition and completion rates |
| 6.92 (1.61) | Conditions that should not be treated with MBSR |
| 6.92 (1.58) | Modality of MBSR (i.e., online versus in person) |
| 6.88 (1.86) | MBSR’s impact on quality of sleep |
| 6.84 (1.97) | Adaptations of MBSR (i.e., for cultural groups and for diagnostic groups, such as perinatal patients or surgical candidates) |
| 6.84 (1.93) | Clinical outcomes of MBSR for youth (e.g., grades, absenteeism, engagement in extracurricular activities, involvement with juvenile justice system, physical health, symptoms of common disorders such as ADHD) |
| 6.84 (1.68) | MBSR’s impact on hypertension |
| 6.84 (1.86) | MBSR’s impact on substance use |
| 6.84 (1.77) | MBSR’s impact on suicidal thoughts and behaviors |
| 6.80 (1.85) | MBSR’s impact on smoking |
| 6.64 (2.02) | Extent to which MBSR addresses social determinants of health (e.g., affordable and safe housing, job attainment, pursuit of education) |
| 6.64 (1.55) | Relative advantage of MBSR coverage options (e.g., claim-based, wellness program, private pay) |
| 6.60 (1.95) | Efficacy of MBSR as prevention/early intervention for youth |
| 6.52 (1.50) | Frequency of MBSR use in the United States (i.e., the Number of people accessing MBSR) |
| 6.52 (1.90) | The demographics of those accessing MBSR |
| 6.40 (1.56) | Frequency of MBSR use in the United States (i.e., the Number of institutions / individuals offering MBSR) |
| 6.32 (1.73) | MBSR’s impact on avoidant behavior |
| 6.12 (1.59 | Group composition of MBSR (e.g., patients and healthcare workers combined versus groups addressing one diagnosis such as cancer) |
| 6.04 (1.79) | MBSR’s impact on preterm birth rates |
| 6.00 (1.83) | MBSR’s impact on symptoms that interfere with successful surgical outcomes |
| 5.88 (1.64) | Frequency of MBSR use in the United States (i.e., the Number of MBSR courses being offered) |
| 5.84 (2.12) | Whether MBSR increases workplace productivity |
| 5.76 (1.51) | How organizational culture and leadership influence MBSR use |

***Note****:* Items with an asterisk (*) were added in Round 2.
